# Depletion of the histone chaperone tNASP inhibits proliferation and induces apoptosis in prostate cancer PC-3 cells

**DOI:** 10.1186/1477-7827-9-50

**Published:** 2011-04-16

**Authors:** Oleg M Alekseev, Richard T Richardson, James K Tsuruta, Michael G O'Rand

**Affiliations:** 1 Department of Cell and Developmental Biology, University of North Carolina, Chapel Hill, NC, 27599, USA; 2 Lineberger Comprehensive Cancer Center, University of North Carolina, Chapel Hill, NC, 27599, USA; 3 Laboratories for Reproductive Biology, University of North Carolina, Chapel Hill, NC, 27599, USA

## Abstract

**Background:**

NASP (Nuclear Autoantigenic Sperm Protein) is a histone chaperone that is present in all dividing cells. NASP has two splice variants: tNASP and sNASP. Only cancer, germ, transformed, and embryonic cells have a high level of expression of the tNASP splice variant. We examined the consequences of tNASP depletion for prostate cancer PC-3 cells.

**Methods:**

tNASP was depleted from prostate cancer PC-3 cells, cervical cancer HeLa cells, and prostate epithelial PWR-1E cells using lentivirus expression of tNASP shRNA. Cell cycle changes were studied by proliferation assay with CFSE labeling and double thymidine synchronization. Gene expression profiles were detected using RT^2^Profiler PCR Array, Western and Northern blotting.

**Results:**

PC-3 and HeLa cells showed inhibited proliferation, increased levels of cyclin-dependant kinase inhibitor p21 protein and apoptosis, whereas non-tumorigenic PWR-1E cells did not. All three cell types showed decreased levels of HSPA2. Supporting in vitro experiments demonstrated that tNASP, but not sNASP is required for activation of HSPA2.

**Conclusions:**

Our results demonstrate that PC-3 and HeLa cancer cells require tNASP to maintain high levels of HSPA2 activity and therefore viability, while PWR-1E cells are unaffected by tNASP depletion. These different cellular responses most likely arise from changes in the interaction between tNASP and HSPA2 and disturbed tNASP chaperoning of linker histones. This study has demonstrated that tNASP is critical for the survival of prostate cancer cells and suggests that targeting tNASP expression can lead to a new approach for prostate cancer treatment.

## Background

Nuclear Autoantigenic Sperm Protein (NASP) is a histone chaperone that binds both core and linker histones [1-4], with a higher affinity for linker histones than core histones [5]; NASP is present in all dividing cells. First characterized in rabbit testis [6] as a homologue to the *Xenopus *oocyte histone binding protein N1/N2 [7,8], NASP has been shown to transport linker histones into the nucleus, transferring H1 histones onto DNA and facilitating chromatin assembly [5]. NASP overexpression [9] as well as NASP depletion induced by siRNA treatment [10] causes disruption in the cell cycle, changes in gene expression profiles [10], and in mice the *NASP*^**-/- **^null mutation is embryonic lethal [11]. Transcribed from a single copy gene, NASP has two splice variants: tNASP, which is found in cancer, transformed, embryonic and germ cells and sNASP, which is found in embryonic and somatic cells [2].

NASP appears to be a multifunctional chaperone protein participating in a variety of regulatory pathways. In developing embryonic stem cells 356 network connectivity episodes have been reported for NASP, suggesting multiple direct protein-protein interactions [12]. During meiosis in the mouse tNASP regulates CDC2/cyclin B1 complex formation through the modulation of HSPA2 ATPase activity [13]; during nucleosome assembly in both DNA synthesis-dependent and independent pathways CAF1 and HIRA are associated with NASP [3,14]; and during DNA repair NASP is associated with KU70 [15].

NASP has been reported as a serologic marker for ovarian cancer, which could be suitable for clinical testing in high-risk populations [16]. Different types of cancer and different stages of the same cancer have been demonstrated to have specific expression profiles for NASP: grade 1 and 2 of breast cancers show up regulation of NASP compared to grade 3 [17]. Estrogen positive tumors express more NASP then estrogen negative ones and similarly sporadic versus BRCA1/BRCA2 mutation positive tumors show different NASP signatures [18]. NASP has become an important constituent of the "poor prognosis signature" in breast cancer patients [19] and the "aggressive tumor gene signature" in lung cancer patients [20]. Although NASP has been reported to be an important prognostic marker in prostate cancer cells [21], it is not clear if tNASP has a specific role is this cancer.

NASP expression is characteristic of all dividing cells, but only cancer, germ, embryonic and transformed cells have a high level of expression of the tNASP splice variant. Consequently we asked the question: what characterizes tNASP in rapidly dividing cells? The current study was undertaken to specifically characterize depletion of tNASP in three different cell lines (prostate cancer PC-3 cells; cervical cancer HeLa cells; non-tumorigenic transformed prostate epithelial PWR-1E cells) and the cellular pathways activated as a consequence.

This study has demonstrated that tNASP is critical for the survival of prostate cancer PC-3 cells and suggests that targeting tNASP expression can lead to a new approach for prostate cancer treatment.

## Methods

### Materials

All chemicals and reagents used in this study were of molecular biology grade. FuGENE^®^6 Transfection Reagent was purchased from Roche Applied Science (Indianapolis, IN). QIAprep Miniprep, QIAquick PCR purification kits, RNeasy mini kit, RNase-Free DNase Set, and Effectene^® ^Transfection Reagent were purchased from Qiagen (Valencia, CA). Restriction enzymes and DNA ligase were obtained from NEB (New England Biolabs, Ipswich, MA). Carboxyfluorescein diacetate, succinimidyl ester (CFSE) and thymidine were purchased from Sigma (Saint Louis, MO). RT^2 ^Profiler PCR Array, RT^2 ^First Strand Kit, and RT^2 ^SYBR Green qPCR Master Mix were purchased from SABiosciences Corporation (Frederick, MD). Sequencing was performed at the University of North Carolina at Chapel Hill automated sequencing facility. Mouse monoclonal anti p53 antibody (DO-1) and rabbit polyclonal anti-p21 antibody (SC-397) were purchased from Santa Cruz Biotechnology (Santa Cruz, CA). Rabbit cleaved caspase-3 (Asp175) antibody was a gift from Dr. Mohanish Deshmukh (Department of Cell and Developmental Biology, University of North Carolina at Chapel Hill). Mouse monoclonal antibody to HSPA2 (ab55290) was purchased from Abcam Inc. (Cambridge, MA). Goat antiserum to full-length recombinant human tNASP (AAH10105) was custom made by Bethyl Laboratories (Montgomery, TX). Goat affinity purified specific anti-tNASP antibody was prepared by absorbing the anti-tNASP antiserum with recombinant sNASP until the anti-tNASP antibody did not react with recombinant sNASP on a Western blot.

### Cell culture

PC-3, HeLa, and PWR-1E [22] cells were used in this study. All the cells were obtained directly from the American Type Culture Collection (ATCC, Manassas, VA).

### Northern blot analysis

Total RNA samples from cell cultures were purified using RNeasy Mini Kit and blotted to Amersham Hybond-N nylon membrane (GE Healthcare, Little Chalfont, UK). The blots were probed with a ^32^P-labeled 750 base pair cDNA from the C-terminal of human NASP, which is identical in sequence for both tNASP and sNASP. Hybridization was carried out in QiuckHyb solution (Agilent Technologies, Santa Clara, CA). Radioactive bands were detected in a PhosphorImager Storm-860 (Molecular Dynamics, Piscataway, NJ).

### Indirect immunofluorescence

Cells were grown on chamber slides with polystyrene wells (BD Biosciences, Bedford, MA), fixed with chilled methanol (-20°C, 20 min) and incubated in goat affinity purified specific anti-tNASP antiserum or pre-immune serum (1:500 in 0.5% BSA in PBS), or in caspase-3 antibody (1:200) for 1 hour. After the washing in PBS twice, cells were incubated in Alexa Flour^® ^488 donkey anti-goat IgG (1:4000 in 0.5% BSA in PBS, Molecular Probes, Eugene, OR). Secondary antibody controls were included in all experiments and were negative.

### Senescence-associated β-galactosidase staining

PC-3, HeLa, and PWR-1E cells were plated on culture slides (BD Biosciences, Bedford, MA) and treated by tNASP shRNA or scrambled shRNA lentivirus. Four days after treatment cells were stained for senescence-activated β-galactosidase activity at pH 6 [23] using a Senescence β-Galactosidase Staining Kit (Cell Signaling Technology, Danvers, MA) accordingly the manufacturer's instructions.

### Double thymidine block

HeLa cells were blocked with thymidine to arrest all the cells at the beginning of S phase [2]. The cells were released by washing out the thymidine, harvested at 0, 4, and 8 hours after release and examined for DNA content by FACS.

### Cell proliferation assay

The cell proliferation (cell division) assay was carried out by labeling scrambled and tNASP shRNA treated PC-3, HeLa, and PWR-1E cells with CFSE [24]. A stock solution (5 mM CFSE in DMSO) was prepared and stored at -80°C. Control and experimentally treated cells were trypsinized, washed twice in sterile PBS and re-suspended in 2 ml of sterile PBS, pre-warmed to 37°C. The stock solution of CFSE was used at a final concentration of 5 μM after incubation at 37°C for 10 min in the dark. To quench the labeling, cells were washed twice with DMEM cell culture medium containing penicillin/streptomycin and 10% FBS. Cells were allowed to grow for an additional 3 days. After 72 hours of growth, cells were washed by PBS, trypsinized, washed in PBS, fixed in 70% ethanol for 2 hours, and stained with tNASP specific antiserum. A parental population of cells was obtained by labeling cells followed by their immediate fixation in 70% ethanol.

### Fluorescent-activated cell sorting (FACS) analysis

Scrambled and tNASP shRNA treated cells 0, 4, and 8 hours after release from the double thymidine block were washed with PBS, trypsinized, and fixed with 70% ethanol for ≥ 2 h on ice. Cells were immunostained with goat anti-tNASP affinity purified antibody, washed twice in PBS and stained with secondary antibody Alexa Fluor 647 donkey anti-goat IgG (H+L) A21447 (Invitrogen, Eugene, OR). Cells were washed in PBS and stained for 30 min at 37°C with 50 μg/ml propidium iodide in PBS (containing 200 μg/ml RNAse A and 0.1% Triton X-100), and incubated overnight at 4°C before analysis at the University of North Carolina at Chapel Hill Flow Cytometry Facility on a Dako Cyan ADP flow cytometer. For each sample at least 10,000 cells were counted. After gating out doublets and debris, cell cycle distribution was analyzed using Summit version 4.3 software (Dako Colorado, Inc., Fort Collins, CO). For the cell proliferation assay, CFSE labeled cells were fixed in 70% ethanol for at least 2 hours and immunostained with specific anti-tNASP antibodies as described above.

### Construction of lentivirus to express shRNA targeting human tNASP

#### Designing shRNA oligos for pLKO.1

For selection of 21-mer targets within the human t-NASP specific part of the sequence (NM_002482), we used an automated service provided by the Broad Institute [25]. Out of more than 200 possible targets 10 top-scoring targets were selected for further study. Forward and reverse oligos compatible with pLKO.1 [26] were designed and produced in the UNC Oligonucleotide Synthesis Core Facility. Annealing of forward and reverse oligonucleotides was carried out in a PCR machine at 95°C for 4 min, 70°C for 10 min with slow cooling to room temperature over 4 hours. The vector pLKO.1 was sequentially digested by Age1 and EcoR1 restriction enzymes and ligated with annealed oligonucleotides using NEB T4 DNA ligase. The ligated construct was propagated in DH5α cells. Plasmids were purified with a Miniprep kit and successful ligations were verified by a sequencing reaction. Individual tNASP shRNA constructs were screened using Effectene^® ^Transfection Reagent for transient knockdown of tNASP mRNA levels in easy to transfect HeLa cells. The two most active shRNA constructs were selected and used for the production of lentiviral particles.

#### Lentiviral particles

Both packaging plasmid psPAX2 and envelope plasmid pMD2.G were obtained from Addgene [27]. Lentiviral particles were generated in HEK-293T cells. Plasmids were transfected using FuGENE^®^6 Transfection Reagent. Target cells were infected by adding lentiviral particles to growth medium. For better transfection polybrene (hexadimethrine bromide, final concentration 8 μg/ml) was added to the culture medium. Infected cells were selected by puromycin pressure added to a final concentration of 3 μg/ml. Negative controls were the mock transfection with Addgene plasmid 1864. The sequence of this shRNA does not have any cellular target [28].

#### RT^2^Profiler PCR Array

RT^2 ^Profiler PCR Array was used as a method of combining real-time PCR performance with a simultaneous analysis of a panel of genes related to the cell cycle (array PAHS-020D. Preparation and analysis of samples were carried out in accordance with the manufacturer's recommendations. Arrays were run on an Opticon 2 thermal cycler (Bio-Rad, Hercules, CA). Threshold cycle values were analyzed using web-based PCR array data analysis software [29]. Reverse transcriptase control, cDNA control, and positive PCR control were within the accepted range.

#### Colorimetric determination of ATPase activity (Malachite Green Assay)

The amount of inorganic phosphate liberated by HSPA2 in the presence of tNASP or sNASP was determined by a malachite green assay [30] based on the formation of a phosphomolybdate complex as we previously described for HIST1H1T [13]. Protein concentrations were 0.5 μM for HSPA2, CDC2, tNASP, sNASP and HIST1H1A. Significance was determined by the Student t-test.

## Results

**Figure 1:**
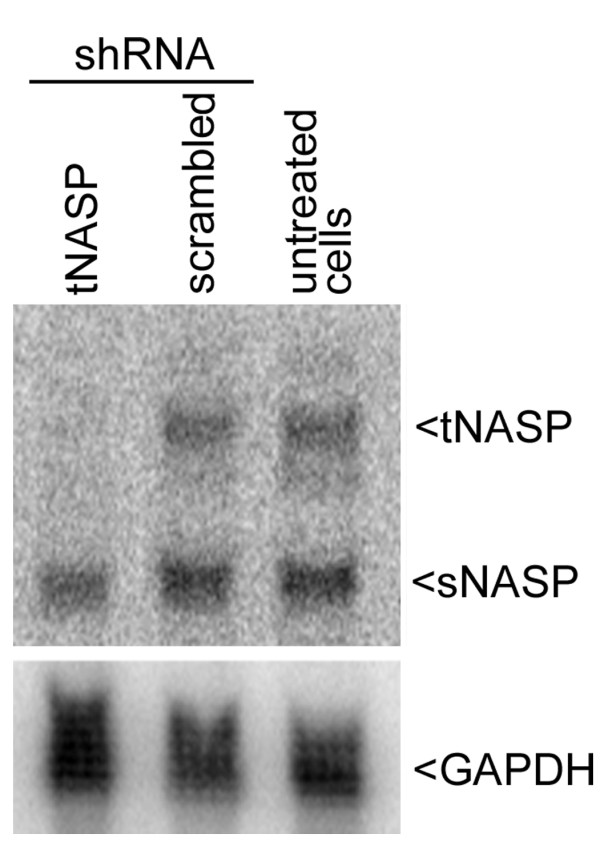
**Northern blot analysis of total RNA from HeLa cells**. Line 1: HeLa cells treated with tNASP shRNA; line 2: HeLa cells treated with scrambled shRNA; line 3: untreated HeLa cells. Blot was hybridized with the 750 bp sequence common to both tNASP and sNASP. Lower panel shows GAPDH loading control. Figure 1 is a representative result from a total of 3 experiments.

**Figure 2:**
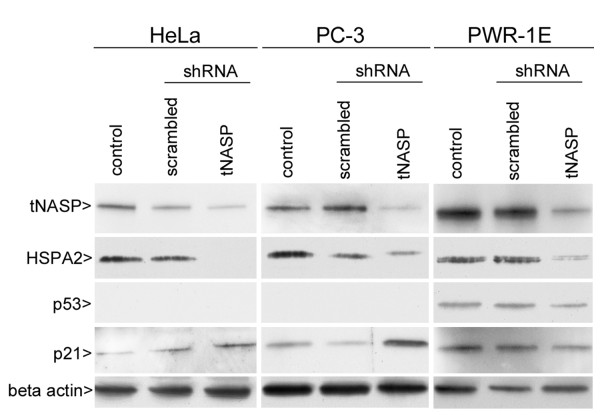
**Western blot analysis of cell lysates after tNASP shRNA treatment demonstrating changes in protein expression in HeLa, PC-3, and PWR-1E cells 6 days after treatment**. Line 1 of each panel: untreated control; line 2 of each panel: scrambled shRNA treatment; line 3 of each panel: tNASP shRNA treatment. Beta actin was used as a loading control. Figure 2 is a representative result from a total of 3 experiments.

**Figure 3:**
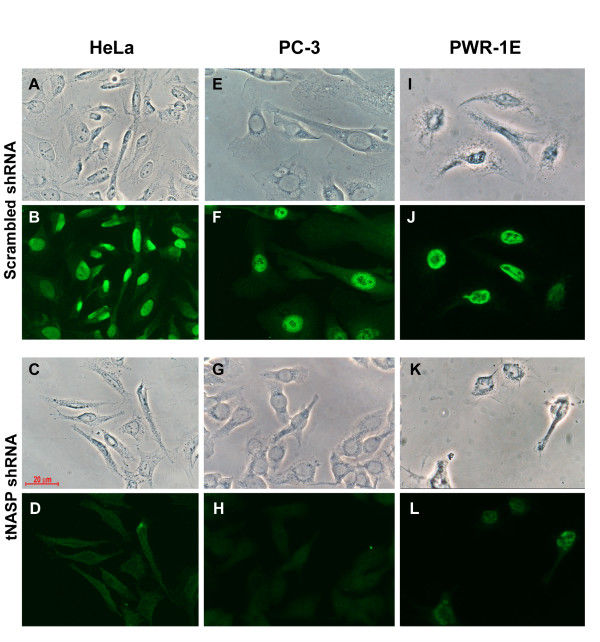
**Localization of tNASP in HeLa, PC-3 and PWR-1E cells**. For each cell type the upper panel shows phase contrast images with the same field below immunostained with anti-tNASP antibody after scrambled shRNA treatment: (A, B) HeLa cells; (E, F) PC-3 cells; (I, J) PWR-1E cells. The lower panel shows phase contrast images with the same field below immunostained with anti tNASP antibody after tNASP shRNA treatment: (C-D) HeLa cells; (G-H) PC-3 cells; (K-L) PWR-1E cells. Scale bar = 20 μm for all panels.

In order to investigate the functions of tNASP we compared three cell lines: the prostate cancer cell line PC-3, the cervix adenocarcinoma cell line HeLa, and the non-tumorigenic prostate epithelial cell line PWR-1E. Initially we found that HeLa cells transiently transfected with tNASP specific shRNA using Effectene lost tNASP mRNA within 4 days after transfection without affecting the mRNA levels of splice variant sNASP (Figure 1). PC-3, HeLa, and PWR-1E cells were treated with lentiviral particles expressing either tNASP shRNA or scrambled shRNA as a negative control and changes in the amount of tNASP protein were examined by Western blotting. While HeLa and PWR-1E cells showed a reduction in tNASP protein after 6 days of treatment, the level of tNASP protein in PC-3 cells by that time was almost undetectable (Figure 2). To determine if the reduction in tNASP protein was the result of a general decrease of tNASP in all cells or a selective depletion in a group of cells, all three cell types were treated with either lentiviral particles expressing tNASP shRNA or scrambled shRNA and immunostained with anti-tNASP antibody 9 days after treatment. HeLa cells were depleted of tNASP in their nuclei (Figure 3C,D) compared to the control (scrambled) treated cells that showed strong nuclear staining for tNASP (Figure 3A,B). A few cells (< 5%) had a strong tNASP signal in the cytoplasm and nucleus. PC-3 cells showed complete depletion of tNASP (Figure 3G,H) in their nuclei whereas control treated PC-3 cells showed strong nuclear staining (Figure 3E,F). PWR-1E cells showed a significant reduction in tNASP, although not to the same extent as HeLa and PC-3 cells. A few PWR-1E cells had tNASP in the cytoplasm and nucleus (Figure 3I,J,K, and 3L). Based on the results of immunostaining, tNASP shRNA treatment efficiently depleted tNASP protein from PC-3, HeLa and PWR-1E cells. However, the level and pattern of depletion was different in the three cell types; most complete depletion was observed in PC-3 cells, while HeLa and PWR-1E showed less effective depletion. The morphology of the tNASP-depleted and tNASP-containing cells based solely on phase contrast microscopy was indistinguishable, except for an increase in dark granules in the cytoplasm of tNASP deficient cells.

### Changes in cell number

**Figure 4:**
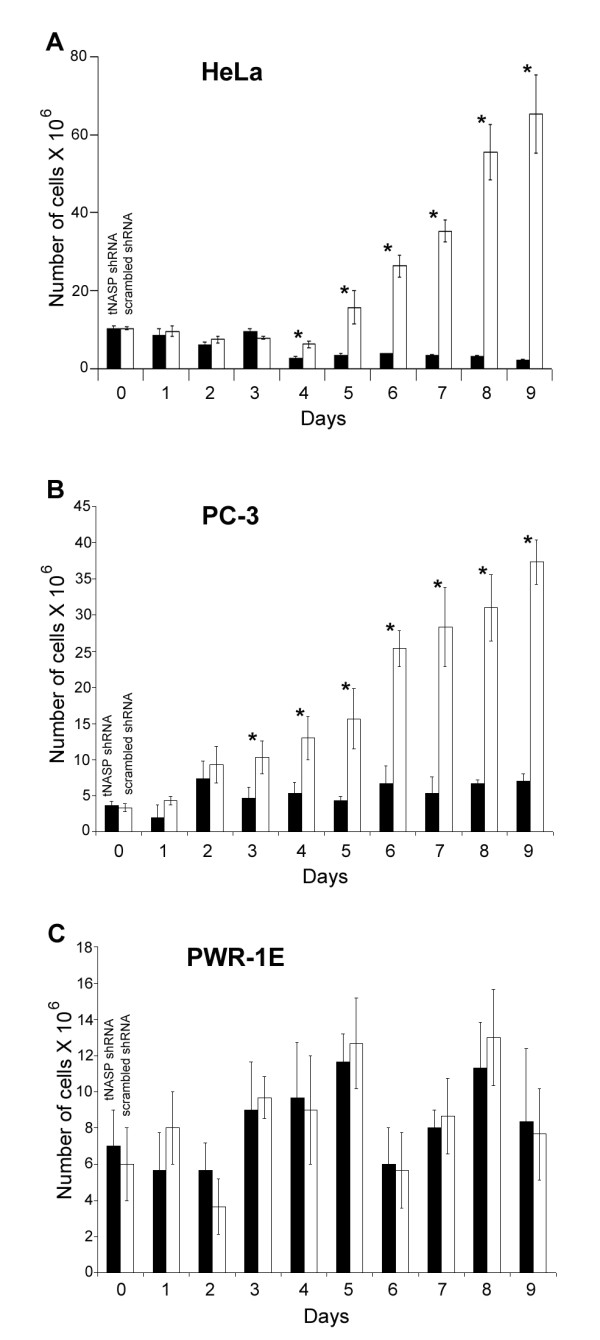
**Dynamic changes in cell number for HeLa, PC-3, and PWR-1E cells treated with tNASP shRNA and scrambled shRNA**. The Y axis indicates the number of cells. The X axis indicates days. The treatment with tNASP shRNA or scrambled shRNA occurred on day 1. The *error bars *represent ± S.D. The asterisks indicate significantly different values (p < 0.05).

To determine if the depletion of tNASP affects cell proliferation and survival, we first studied the changes in cell numbers. As shown in Figure 4, the three cell lines were treated with either lentiviral particles expressing tNASP shRNA or scrambled shRNA on day 1. During the first 2-3 days neither treatment group showed any increase in the cell numbers, which was probably a reflection of the general cytotoxicity of lentiviral and puromycin selection treatments. Starting on day 5 for HeLa cells (Figure 4A) and day 3 for PC-3 cells (Figure 4B) a difference between experimental and control treated cells became apparent in that the number of control treated cells began to increase, while the number of experimentally treated cells remained relatively unchanged. In striking contrast, the number of PWR-1E cells in the experimental and control groups did not differ significantly from each other, although generally the number of cells in the control group was higher. The fluctuations in PWR-1E cell numbers were probably due to their increased sensitivity to growth conditions [31]. As a result of these experiments we asked whether the decline in the number of cells was due to cell death or was the result of a decreased proliferation rate.

### Affect of tNASP depletion on cell proliferation

**Figure 5:**
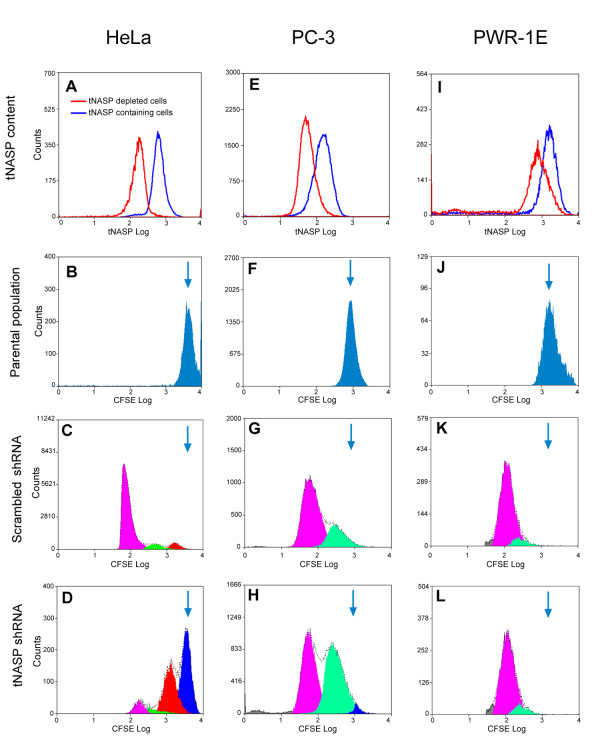
**Depletion of tNASP from HeLa and PC-3 cells decreases cell proliferation**. Upper panel "tNASP content" (A, E, I) shows the distribution of tNASP depleted and tNASP containing cells after the tNASP shRNA treatment as determined by FASC analysis on day 6. Panel "Parental population" (B, F, J) shows the original position of labeled, non-dividing cells on day 3. Panels "Scrambled shRNA" (C, G, K) and "tNASP shRNA" (D, H, L) show the proliferation of cells after they were treated by tNASP shRNA or scrambled shRNA. Each peak shows the population of cells with diminished content of CFSE due to cell divisions. Data were collected on day 6. Only tNASP depleted cells were analyzed for proliferation, tNASP containing cells were gated out. Figure 5 is a representative result from a total of 3 experiments.

As described above (Figure 3), there was some variation among individual cells within a cell line with regard to the amount of tNASP decrease as a result of tNASP shRNA lentiviral treatment. Using anti-tNASP antibodies we determined by FACS the distribution of tNASP-positive and tNASP-negative cells on day 6 (upper panel, Figure 5A,E,I). In every cell population there were cells depleted of tNASP (red line histogram) and tNASP containing cells (blue line histogram). The highest number of tNASP depleted cells was observed in the PC-3 cell sample, corresponding to the observations from immunostaining (Figure 3).

Only the tNASP depleted cells were gated in the following FACS experiments and used for the proliferation assay. To determine the position of undivided cells (called the parental population) a portion of the PC-3, HeLa, and PWR-1E cells was labeled with CFSE, a protein stain, after tNASP shRNA treatment and immediately fixed. The CFSE labeling in each group of cells is shown as the parental population in Figure 5B,F, and 5J and marks the position of the original amount of CFSE in each cell line. The experimental and control transfected cells were labeled with CFSE on day 3 after treatment and grown for an additional 3 days. Control HeLa cells (Figure 5C) show three distinct peaks (from arrow, right to left in Figure 5C) representing three cell division cycles with no cells remaining undivided (the amount of CFSE in all 3 peaks is equal to that in the original parent population) and 81% of the cells completed the third division. In contrast 48% of tNASP shRNA treated HeLa cells could not complete the first division (Figure 5D), 34% completed the first division and only 18% could complete the second and third divisions.

Control treated PC-3 cells examined under identical experimental conditions to the HeLa cells completed either one or two divisions by day 3 (Figure 5G): 71% completed two divisions, 28% completed one division, and 1% did not divide. In contrast, proliferation was significantly affected in tNASP shRNA treated cells: 46% completed two divisions, 51% completed only one division, and 3% of cells did not divide (Figure 5H). PWR-1E cells were distinct from both HeLa and PC-3, under identical conditions they completed 2 divisions and there was no difference between experimental and control (scrambled) treated cells (Figure 5K,L). These experiments demonstrate that depletion of tNASP has distinct effects on different cell lines, retarding cell cycle proliferation in PC-3 and HeLa cells but not in PWR-1E cells.

### Cell cycle progression

**Figure 6:**
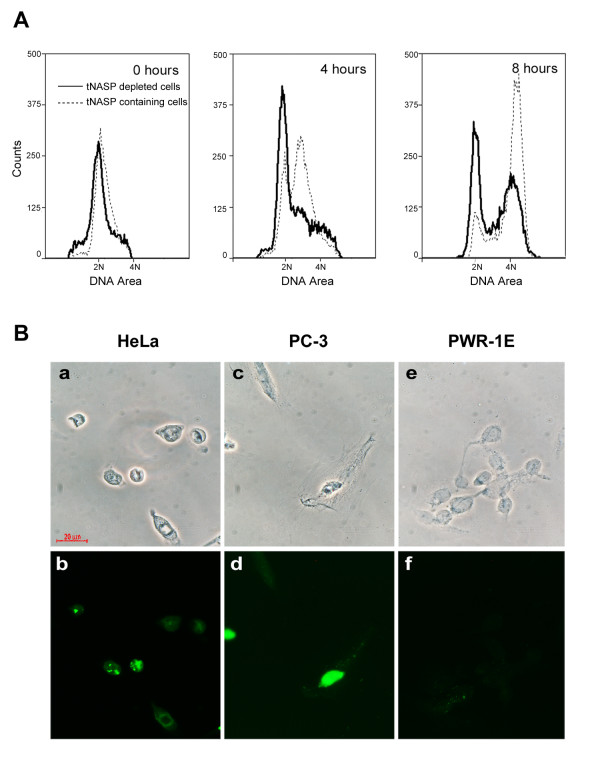
**tNASP depleted cells slow their progression through the cell cycle and develop apoptosis markers**. **A**. FACS analysis demonstrating double thymidine-blocked HeLa cells allowed to progress in synchrony through the cell cycle. Solid line: HeLa cell progression through the cell cycle after treatment with tNASP shRNA; Dotted line: HeLa cell progression after treatment with scrambled shRNA. **B**. Immunostaining for caspase-3 in HeLa, PC-3, and PWR-1E cells after treatment with tNASP shRNA. Scale bar = 20 μm for all panels. Upper panel shows phase contrast images and lower panel shows corresponding immunostaining for caspase-3. HeLa cells (a, b); PC-3 cells (c, d); PWR-1E cells (e, f). Control caspase-3 staining of scrambled shRNA treated cells was negative for all samples (data not shown).

We next determined the phase of the cell cycle in which the proliferation of tNASP depleted cells was blocked. As shown in Figure 6A, tNASP shRNA (solid line) and control (scrambled, dotted line) treated HeLa cells were synchronized by a double thymidine block and observed for their progression through the cell cycle. As in the proliferation assay (Figure 5) only tNASP depleted cells were gated in the FACS experiments. At the time of release (Figure 6, 0 hours panel) from the double thymidine block both groups of cells looked identical; 78.5% of tNASP shRNA treated cells and 83.3% of scrambled shRNA treated cells were in G_1 _phase. After 8 hours more than 80% of control treated cells replicated their DNA and moved to the G_2 _phase, however less than 50% of tNASP shRNA treated cells moved to the G_2 _phase. 42.9% of tNASP shRNA treated cells remained in G_1 _phase, compared to 13% of the control treated cells (Figure 6; 8 hours panel). These data indicate that cells depleted of tNASP are held in the G_1 _phase.

### Senescence or apoptosis

We next asked the question: what happens to the tNASP depleted PC-3 and HeLa cells? To determine if the cells undergo senescence we tested for β-galactosidase activity at pH 6, which is a known characteristic of senescent cells [32]. PC-3, HeLa, and PWR-1E tNASP depleted cells did not show any β-galactosidase activity (data not shown). To determine if the cells undergo apoptosis we stained the tNASP depleted cells for active caspase-3, which is a marker for early apoptosis and indicative of cells in the apoptosis pathway [33,34]. Four days after tNASP shRNA treatment PC-3, HeLa and PWR-1E cells were immunostained with antibody to cleaved caspase-3. As shown in Figure 6B staining reveled that numerous tNASP depleted PC-3 and HeLa cells contained activated caspase-3 (Figure 6B; a-b; c-d). Control treated cells were negative for activated caspase-3 (data not shown). In contrast to PC-3 and HeLa cells, tNASP depleted PWR-1E cells were negative for caspase-3 (Figure 6B; e-f). These data indicate that in PC-3 and HeLa cells the depletion of tNASP induces apoptosis, in contrast to non-tumorigenic PWR-1E cells in which proliferation is unaffected and apoptosis is not induced.

### Gene expression profiles

Because NASP is a multifunctional chaperone protein that may interact with a variety of other proteins we analyzed the gene expression profiles by real time PCR using RT^2 ^PCR arrays in PC-3, HeLa, and PWR-1E cells depleted of tNASP. Experimental and control treated cells were sampled four days after treatment when the difference in cell number first becomes apparent. Genes that were up or down regulated by at least two fold are shown in Table 1. Cyclin-dependant kinase inhibitor 1A (p21) was the only up regulated gene common to all three cell types. Additionally HeLa and PWR-1E cells showed a common up regulation of cyclin-dependant kinase 6. There were no common down regulated genes, although cyclin D1 and cyclin D2 were down regulated in PC-3 and PWR-1E cells respectively.

**Table 1:** Cell cycle related genes regulated by the depletion of tNASP

**Gene name**^**1**^	**Gene Symbol (GenBank)**^**2**^	Fold change
HeLa cells		
Cyclin-dependent kinase inhibitor 1A (p21)	CDKN1A (NM_000389)	3.67
Cyclin-dependent kinase 6	CDK6 (NM_001259)	2.69
PC-3 cells		
Cyclin D2	CCND2 (NM_001759)	12.45
Cyclin-dependent kinase inhibitor 2A (p16)	CDKN1A (NM_000389)	2.87
Beta-2-microglobulin	B2M (NM_004048)	2.52
Cyclin G2	CCNG2 (NM_004354)	2.46
Cyclin-dependent kinase inhibitor 1A (p21)	CDKN2A (NM_000077)	2.45
Cyclin B2	CCNB2 (NM_004701)	2.4
HUS1checkpoint homolog	HUS1(NM_004507)	-2.15
Cullin2	CUL2 (NM_003591)	-2.6
Cyclin D1	CCND1 (NM_053056)	-9.69
PWR-1E		
CDC28protein kinase regulatory subunit 2	CKS2 (NM_001827)	4.38
Cyclin-dependent kinase inhibitor 1A (p21)	CDKN1A(NM_000389)	2.45
Cyclin G2	CCNG2 (NM_004354)	2.42
Cyclin-dependent kinase inhibitor 1B (p27)	CDKN1B (NM_004064)	2.40
Cyclin-dependent kinase 6	CDK6 (NM_001259)	2.25
Cyclin F	CCNF (NM_001761)	2.24
Cyclin D2	CCND2 (NM_001759)	-2.38
B-cell Cell/lymphoma 2	BCL2 (NM_000633)	-2.83

^1 ^Genes listed present those differentially regulated after HeLa, PC3, RWPE-2, and PWR-1E cells were infected by lentiviral particles containing tNASP shRNA as compared to cells infected with scramble shRNA. The cutoff limit in the analysis was set to two-fold for both induced (+) and repressed (-) genes.

^2 ^Gene Bank accession number

### Expression of p21

Expression of p21 was confirmed by Western blots six days after treatment. As shown in Figure 2, p21 is up regulated in PC-3 and HeLa cells but not in PWR-1E cells. The protein p21 is a well established inhibitor of cyclin-CDK2 or cyclin-CDK4 complexes and functions as a regulator of cell cycle progression at G_1_, consistent with our findings in the cell cycle analysis.

### HSPA2 activity

**Figure 7:**
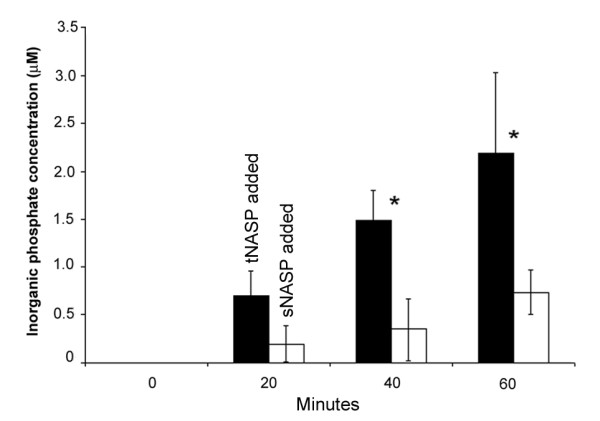
**tNASP, not sNASP activates the ATPase activity of HSPA2**. ATPase activity of HSPA2 is presented as inorganic phosphate (μM) liberated by HSPA2 within 60 min of incubation at 37°C in the presence of tNASP or sNASP, CDC2, and histone H1a. Data are represented as mean ± SD. The asterisks indicate significantly different values (p < 0.05). Figure 7 is a representative result from a total of 3 experiments.

To elucidate the mechanism of anti-proliferative and pro-apoptotic effects of tNASP depletion in prostate cancer cells we examined HSPA2 ATPase activity. HSPA2 is required for the survival of cancer cells; HSPA2 depletion has been reported to arrest cells in G_1 _and up regulate p21 expression [35]. However, non-tumorigenic cell lines appear to be unaffected by depletion of HSPA2 [36]. Previously we reported that in mouse spermatogenic cells HSPA2 ATPase activity increased after interaction with tNASP in the presence of linker histone H1t, cyclin B1 and CDC2 [13]. Using an identical experimental paradigm we compared the *in vitro *modulation of HSPA2 ATPase activity by tNASP or sNASP in the presence of cyclin B1, CDC2 and histone H1a. Histone H1a was selected because it is the linker histone with the highest level of expression in somatic cells [37]. As shown in Figure 7 we found that HSPA2 was three times more active in the presence of tNASP than in the presence of sNASP. These results are consistent with PC-3 and HeLa cells requiring tNASP to maintain high levels of HSPA2 activity to remain viable while PWR-1E cells remain viable and are insensitive to lower levels of HSPA2 activity.

## Discussion

This study has demonstrated that selective depletion of tNASP from PC-3, HeLa and PWR-1E cell lines in culture leaves sNASP unaffected and results in distinctly different effects. Using lentiviral expression of tNASP shRNA, all three cell lines had decreased HSPA2 protein levels, yet only PC-3 and HeLa cells increased expression of p21 and entered apoptosis. NASP has been detected in all dividing cells, but only cancer, transformed, germ, and embryonic cells have a high level of expression of the tNASP splice variant [2]. Depletion of both tNASP and sNASP has been shown to be embryonic lethal and without sufficient NASP HeLa and U2OS cells were unable to enter S phase [11]. However, these studies did not distinguish whether tNASP and sNASP played separate roles and raised the question why rapidly dividing cells such as cancer or embryonic cells expressed tNASP while adult somatic cells did not.

The results reported in this study clearly demonstrated that in prostate cancer PC-3 cells and cervical cancer HeLa cells depletion of tNASP caused significant anti-proliferative effects and induced apoptosis while the non-tumorigenic prostate epithelial PWR-1E cells did not experience these changes in the time span observed. Cells depleted of tNASP slowed down their proliferation and were blocked at the G_1_/S border of the cell cycle, which is consistent with our earlier report [11].

The activation of p53 could result in apoptosis, cell-cycle arrest, DNA repair or senescence [38,39]; however activation of p53 is not relevant in our study because we used cell lines with non-functional, inhibited or mutated p53. HeLa cells, which originate from adenocarcinoma of the cervix, contain wild-type p53 alleles together with the DNA of specific types of human papillomaviruses [40] and the transcriptional stimulatory activity of HeLa cell p53 is strongly repressed by one of the oncogenic papilomavirus products, E6 protein [41]. In PC-3 prostate cancer cells p53 is absent due to deletion of a base pair in the single copy gene and that generates a frame shift and a new immediate stop codon [42,43]. PWR-1E cells were immortalized by adenovirus 12-SV40 and although they show strong nuclear staining for p53 [22] its activity is inhibited by the binding between p53 and SV40 Large T-antigen [44]. In this study p53 protein could only be detected on Western blots in PWR-1E cells (Figure 2).

The up regulation of p21 appears to be important in the cell lines we studied. Expression of p21 mRNA increased in all three cell lines; however protein levels were increased only in PC-3 and HeLa cells. p21 is known to be a potent cell cycle progression inhibitor because it inhibits the activity of cyclin-dependant kinase-cyclins A, D, and E complexes [45]. Although it is well established that p21 mediates p53-dependant G_1_/S phase growth arrest in response to DNA damage [46,47], p21 expression could be induced by mechanisms independent of p53. In this case the expression of p21 is up regulated by different transcription factors: E2F1 through the activation of Ras [48], SP1, SP3, AP2, HOXA10, STAT [49], AP-1 [50].

Because tNASP is a chaperone for linker histones and transports them into the nucleus in an energy and nuclear localization signal-dependent manner [51], the depletion of tNASP implies that there could be a simultaneous depletion of H1 histones in the nucleus. Depletion of linker histones has been reported to be sufficient for altering the expression of subsets of genes, the up regulation of p21 in particular, and for G_1_-phase cell cycle arrest in human breast cancer cells [52]. Regulation of DNA replication through multiple pathways in egg extracts has been reported to be dependent on sufficient linker histones [53]. Decreased levels of H1 histones down to 50% from their normal level caused profound chromatin structural changes, including global nucleosome spacing, reduced chromatin compaction, and decreases in core histone modifications [54]. Moreover, linker histones are critical for the interaction of tNASP and HSPA2. We previously reported [13] that in mouse spermatogenic cells tNASP in the presence of CDC2 requires H1 linker histones to significantly increase HSPA2 ATPase activity. Therefore, depletion of tNASP should result in lesser amounts of HSPA2 activity implying that the interaction of tNASP and HSPA2 may be an important factor in the differential response of cancer cell lines to the depletion of tNASP. All three cell types in this study showed a decrease in HSPA2 protein levels (Figure 2) and the ATPase activity of HSPA2 was significantly increased in the presence of tNASP, but not sNASP (Figure 7).

HSPA2 activity was previously reported as a necessary chaperone for formation of an active CDC2-cyclin B1 complex in pachytene spermatocytes as a condition for successful completion of the G_2_/M transition [55]. HSPA2 is not only essential for spermatogenesis [56], but is required for tumor cell growth and survival [57]. HSPA2 depletion resulted in a senescence-like condition, arrest in the G_1 _phase of the cell cycle and up or down regulation of genes such as MIC-1 and p21 [35]. Interestingly HSPA2 is required for survival of cancer cells [35], while non-tumorigenic cells (mammary MCF-10A, HBL-100, and prostate PNT1A) displayed no changes in survival after depletion of HSPA2. This selective effect is similar to our observation that although PWR-1E cells lost HSPA2 protein their survival was not affected.

## Conclusions

Based on our data we conclude that the effect of tNASP depletion could be signaled through at least two different pathways: a loss of HSPA2 activity and a shortage of linker or core histones. Either of these pathways might lead to inhibited proliferation and apoptosis. The data presented here demonstrate that tNASP is required for the survival of prostate cancer PC-3 cells and its depletion results in anti-proliferative and pro-apoptotic activity. Targeting tNASP expression could lead to a new approach for cancer treatment.

## List of abbreviations

The abbreviation used are: H1t: testis specific linker histone; NASP: nuclear autoantigenic sperm protein; tNASP: testis/embryo form of nuclear autoantigenic sperm protein; sNASP: somatic/embryo form of nuclear autoantigenic sperm protein; CAF: chromatin assembly factor; HIRA: histone cell cycle regulation defective homolog A; HSPA2: heat shock protein A2; H1a: linker histone a; BSA: bovine serum albumin; PBS: phosphate buffered saline.

## Competing interests

The authors declare that they have no competing interests.

## Authors' contributions

OMA conceived of the study, carried out the molecular biologic studies, FACS and RT^2 ^Profiler PCR Array data analysis, and drafted the manuscript. RTR assisted with experimental design and has been involved in drafting the manuscript. JKT made substantial contributions to acquisition of data and critical revision of manuscript. MGO conceived of the study, participated in project design and coordination, helped to draft the manuscript. All authors read and approved the final manuscript.
